# Impact of Early Percutaneous Coronary Intervention on Long-Term Survival in Patients With Acute Myocardial Infarction

**DOI:** 10.7759/cureus.101145

**Published:** 2026-01-09

**Authors:** Khurrum Rashid, Uzair Ullah Qureshi, Hafiz Ali Shabbir Rajput, Imadud Din, Abbas Anwar, Muhammad Hamza Ali khan, Usman Baig, Anielka Cristina C Bonilla Arauz, Saifullah Chan

**Affiliations:** 1 Department of Internal Medicine, Ayub Medical College, Abbottabad, PAK; 2 Department of Cardiology, Abbottabad International Medical Institute, Abbotabad, PAK; 3 Department of Urology, Royal Liverpool University Hospital, Liverpool, GBR; 4 Department of Internal Medicine, Khyber Medical University, Peshawar, PAK; 5 Department of Internal Medicine, Abbottabad International Medical Institute, Abbottabad, PAK; 6 Department of General Medicine, Universidad Nacional Autonoma de Nicaragua- Leon, Leon, NIC; 7 Department of Internal Medicine, Ayub Teaching Hospital, Abbotabad, PAK

**Keywords:** acute myocardial infarction, heart failure, long-term outcome, percutaneous coronary intervention, reperfusion

## Abstract

Background: Early percutaneous coronary intervention (PCI) is the recommended standard of care for acute myocardial infarction (AMI), but long-term outcomes in mixed real-world cohorts remain underreported. This study evaluated the effects of early PCI (≤24 hours) compared with delayed or no PCI on short- and long-term clinical outcomes.

Materials and methods: A five-year mixed cohort study was conducted and included 891 consecutive AMI patients (early PCI, n = 446; delayed/no PCI, n = 445). Demographics, clinical characteristics, procedural data, and in-hospital outcomes were collected. Long-term outcomes, such as all-cause mortality, cardiovascular mortality, recurrent myocardial infarction (MI), heart failure (HF) hospitalization, and major adverse cardiovascular events (MACE), were assessed over a median follow-up of 48 months. Propensity score matching and Cox proportional hazards models were used to adjust for confounding. Statistical analyses were done in the IBM SPSS Statistics software, version 27.0 (IBM Corp., Armonk, NY, USA).

Results: Early PCI was associated with lower in-hospital mortality (18/446, 4.0% vs 35/445, 7.9%; p = 0.01), shorter door-to-balloon time (median 65 vs 210 minutes; p < 0.001), and better left ventricular function (mean left ventricular ejection fraction (LVEF) 48.7% vs 46.2%; p < 0.001). Over a median follow-up of 48 months, early PCI significantly reduced all-cause mortality (62/446, 13.9% vs 112/445, 25.2%; adjusted hazard ratio (HR) 0.54, 95% CI 0.40-0.73, p < 0.001), cardiovascular mortality (44/446, 9.9% vs 82/445, 18.4%; adjusted HR 0.53, 95% CI 0.37-0.77, p = 0.001), HF hospitalization (56/446, 12.6% vs 84/445, 18.9%; adjusted HR 0.66, 95% CI 0.47-0.93, p = 0.02), and MACE (92/446, 20.6% vs 138/445, 31.0%; adjusted HR 0.63, 95% CI 0.49-0.82, p < 0.001). Recurrent MI was slightly lower with early PCI (38/446, 8.5% vs 49/445, 11.0%; adjusted HR 0.78, 95% CI 0.52-1.16, p = 0.21) but did not reach statistical significance.

Conclusion: Early PCI confers substantial short- and long-term survival benefits in AMI patients, with significant reductions in all-cause and cardiovascular mortality, HF hospitalization, and MACE. These findings underscore the critical importance of timely reperfusion, supporting guideline-driven early PCI strategies in real-world practice.

## Introduction

Acute myocardial infarction (AMI) is one of the primary causes of morbidity and mortality worldwide, with a very large healthcare burden and premature death [1]. Although medical treatment and management of the risk factors have advanced, the capacity to reestablish coronary flow of blood in a timely manner is essential in lessening the degree of cardiac damage and enhancing survival among the patients. Percutaneous coronary intervention (PCI) has become the pillar of reperfusion therapy in ACS, showing definite decreases in short-term and long-term adverse cardiovascular events [2,3]. Early intervention has been demonstrated to improve left ventricular function, minimize the risk of heart failure (HF), and reduce mortality, preferably within the initial 24 hours of symptom onset, in several clinical trials and registries [4].

The pathophysiological rationale of the early PCI benefits is based on the reduction of ischemic time, the decrease of infarct size, and the availability of viable myocardium. Research has always demonstrated that prompt reperfusion substantially reduces hospital mortality and significant adverse cardiovascular events (MACE) as opposed to delayed intervention [5, 6, 7]. Furthermore, prompt PCI has been associated with fewer complications (cardiogenic shock, arrhythmias) [8], and hence the significance of prompt patient triage and procedural preparedness. Although these advantages are well-established, differences in practice trends, healthcare systems, and timing of presentation of patients can also lead to delays in intervention, especially in countries with middle or low incomes.

The long-term benefits of early PCI have also been highlighted by observational studies [9], review articles [10], and meta-analyses [11], and such research has shown sustained results in mortality from cardiovascular disease, repeated myocardial infarction (MI), and HF hospitalization in the long follow-up periods. Noteworthy, these advantages have been found in diverse patient subgroups, including elderly patients, patients with illnesses (including diabetes or hypertension), and patients with higher Killip classes at presentation. Nevertheless, most of the available evidence stems from Western populations, and evidence from South Asian regions is relatively scarce.

Although within the guidelines, the recommendation to administer PCI during the initial 24 hours following disease start to patients eligible to receive this treatment encourages timely recovery, real-world issues such as the delay to go to a catheterization facility, the lack of a catheterization facility around the patient, and the resources needed to deliver timely PCI tend to interfere with achieving timely recovery [12,13]. This gap between care practice guided by guidelines and clinical reality supports the necessity of region-specific research that would evaluate both the outcomes of the procedures and the population's continued existence in locations where healthcare was previously inferior to that of high-resource areas.

Understanding the effects of early PCI is particularly important in settings where cardiovascular risk factors are prevalent and delays in care are common. Even modest reductions in time-to-reperfusion can translate into meaningful improvements in survival and quality of life, as suggested by previous studies. Evaluating both short-term in-hospital outcomes and long-term cardiovascular events enables clinicians and policymakers to identify gaps in care delivery, optimize treatment strategies, and maximize patient benefit.

Cardiovascular disease is an emerging health challenge in Khyber Pakhtunkhwa (KPK), Pakistan. Limited access to specialized cardiac care centers, geographic barriers, and delays in patient presentation contribute to increased morbidity and mortality from AMI in the region. Although PCI has become a widely recognized intervention, there remains a scarcity of data on the timing, efficacy, and long-term effects of the procedure in this population. Addressing this gap is crucial to improving local healthcare outcomes and supporting evidence-based practice. The present study aimed to assess the impact of early PCI on long-term survival among patients with AMI in a mixed cohort from KPK, Pakistan. We examined both in-hospital and long-term outcomes to provide region-specific insights into the benefits of timely reperfusion on patient survival.

## Materials and methods

Study design and setting

This five-year mixed cohort study followed patients placed in the tertiary care hospital, Ayub Teaching Hospital in Abbottabad, Pakistan, from November 1, 2020, to October 31, 2025, with a three-year retrospective and approximately two-year prospective follow-up period. The purpose of the study was to investigate the impact of early PCI on the long-term survival of patients with AMI.

Retrospective data were derived based on hospital electronic medical records, cardiac catheterization laboratory databases, and the national mortality registry. Standardized telephone interviews of prospective follow-up data and predetermined outpatient questionnaires were further used to gather prospective data to guarantee full ascertainment of survival.

This mixed design was selected to achieve the highest statistical power and gain the most events in a reasonable period of time to be able to assess both past results and future survival curves in a modern AMI population. The study has followed the Strengthening the Reporting of Observational Studies in Epidemiology (STROBE) template of observational research [14].

Study population

The eligibility criteria included all adult patients with a primary diagnosis of AMI and admitted within the study period (18 years and older). The diagnosis of AMI relied on the Fourth Universal Definition of Myocardial Infarction (2018) that states ACS requires an increase and/or a decrease in any cardiac troponin, at least one of which should exceed the 99^th^ percentile of the upper limit of reference, in combination with clinical risk of myocardial ischemia, as evidenced by signs of ischemic symptoms, electrocardiographic changes, imaging findings, or a coronary thrombus present [15].

Patients were not included in case of type 2 MI, transfer since a previous PCI, having undergone emergent coronary artery bypass grafting (CABG) prior to any attempted PCI, terminal non-cardiac sickness with a life expectancy of fewer than six months, and insufficient verifiable follow-up data.

Sample size and power justification

The hazard ratio (HR) represents the expected relative risk of adverse outcomes in the early PCI group compared with the delayed/no PCI group, and π\piπ is the proportion of participants allocated to each group. Assuming a two-sided significance level of α=0.05, 80% power (1−β=0.8), 1:1 allocation (π=0.5), and a baseline five-year survival of 80% (20% event rate) in the delayed/no PCI group, the total sample size required depends on the expected effect size (HR). To find a difference in long-term survival, the sample size was calculated between early PCI (≤24 hours) and delayed/no PCI groups using a log-rank test based on the proportional hazards assumption. Calculations were performed using the Freedman formula for survival analysis:

$\frac{(Z_{1-\alpha/2} + Z_{1-\beta})^{2}} {\left( \ln(\mathrm{HR}) \right)^{2}\, \pi (1-\pi)}$

where n = total number of participants required; HR = assumed hazard ratio comparing early PCI to delayed/no PCI; π = proportion of participants allocated to each group (e.g., 0.5 for 1:1 allocation); Z_1−α/2_ = Z-score corresponding to the two-sided significance level α (e.g., 1.96 for α=0.05); Z_1−β_ = Z-score corresponding to the desired statistical power 1−β (e.g., 0.84 for 80% power); α = Type I error rate (probability of false positive); and β = Type II error rate (probability of false negative).

For an anticipated HR of 0.65, a clinically meaningful reduction supported by prior observational evidence, the formula yields a required total sample of approximately 846 participants. Accounting for an anticipated 5% loss to follow-up (based on registry linkage and active tracking procedures), the final inflated target sample becomes ≈ 891 participants, providing a conservative and feasible range of 850-900 patients.

This target remains adequately powered to detect the prespecified effect size (HR ≈ 0.65) under the stated assumptions. The proposed sample size is consistent with comparable contemporary studies assessing long-term outcomes after PCI or CABG. For example, Chang et al. [16] analyzed 865 patients with unprotected left main bifurcation disease; Nammas et al. [17] randomized 827 ACS patients with seven-year follow-up; Tokuda et al. [18] studied 402 nonagenarian PCI patients; and Jonik et al. [19] compared 267 patients with lateral left main coronary artery disease undergoing PCI or CABG. These cohort sizes and follow-up durations support the adequacy and realism of the present study’s sample size.

Exposure definition

The key exposure variable was the time of PCI. Early PCI was defined as intervention performed within 24 hours of hospital arrival, consistent with contemporary European Society of Cardiology (ESC) and American College of Cardiology/American Heart Association (ACC/AHA) guidelines [20,21]. In a secondary sensitivity analysis, PCI carried out within 12 hours was referred to as early PCI. The exact time of PCI was determined from catheterization laboratory timestamps. Patients were categorized into three groups based on timing of revascularization: Early PCI, defined as PCI performed within 24 hours of hospital arrival; Delayed PCI, defined as PCI performed more than 24 hours after hospital arrival during the index hospitalization; and No PCI, defined as patients who did not undergo PCI during the index hospitalization. The timing of PCI was determined using catheterization laboratory timestamps.

Outcome measures

The primary outcome was long-term all-cause mortality, calculated from the time of hospital admission to death or last known follow-up. Secondary outcomes included cardiovascular mortality, hospital readmission due to HF, and a composite of MACE, defined as urgent repeat revascularization, recurrent myocardial infarction, or all-cause death [22].

Outcomes were initially assessed and reported across three predefined PCI exposure groups: early PCI (≤24 hours), delayed PCI (>24 hours during index hospitalization), and no PCI. For the primary comparative analysis, outcomes in the delayed PCI and no-PCI groups were additionally combined and compared with the early PCI group to evaluate the impact of early revascularization on long-term prognosis. Whenever available, causes of death were verified using official death certificates and hospital medical records.

Follow-up procedures

In the case of all patients, the time was tracked starting with the index hospitalization up to death or loss to follow-up or the termination of the study (December 31, 2023). Follow-up was done using various complementary ideas such as hospital readmission data, outpatient clinic data, and connection with the national mortality database. Moreover, research nurses who are trained were interviewed annually via telephone through a structured questionnaire. Those patients who could not be contacted via phone were contacted via mail or using other family contacts. Median follow-up time was noted, and those patients who were not contacted after following up numerous times were censored to the date of the last contact.

Questionnaire component

To get patient-reported outcomes, functional status, and lifestyle behaviors on long-term follow-up, a structured questionnaire has been designed (Appendix A). The questionnaire was filled in by trained personnel who had no idea of the time of PCI to prevent interview bias. It had cardiovascular health (recurrence of angina, hospital readmission, medication compliance), functional status using the Canadian Cardiovascular Society (CCS) Angina Classification [23], and quality of life based on a five-point Likert scale addressing physical limitation, anxiety, and social participation. Self-reported information on lifestyle change, particularly smoking cessation, diet, and exercise, was used to assess lifestyle modification. Hospital records were checked against all the reported cardiovascular events to validate them and reduce the effect of recall bias.

Data collection and covariates

Hospital admission records were used to collect baseline data, which comprised demographic (age, sex), cardiovascular risk factors (hypertension, diabetes mellitus, lipid abnormalities, smoking status), and previous cardiovascular disease events (previous MI, PCI, or CABG). Presentation clinical characteristics, time since the start of the symptoms to presentation at the hospital, Killip classification, systolic blood pressure, and heart rate were also noted.

Laboratory and imaging data involved serum creatinine, peak troponin, and left ventricular ejection force through echocardiography. Culprit vessel, type of stent, door-to-balloon time, and intra-procedural complications were data that were gathered through catheterization laboratory databases.

Trained clinical data officers abstracted all the data using standardized definitions and data forms. To verify the accuracy of the data, the second auditor randomly sampled 10% of the records and agreed on the discrepancies.

Statistical analysis

Statistical analyses were performed using IBM SPSS Statistics software for Windows, version 27.0 (IBM Corp., Armonk, NY, USA). Continuous variables were summarized as means with standard deviations (SD) or medians with interquartile ranges (IQR), as appropriate, while categorical variables were presented as frequencies and percentages. Baseline characteristics were initially compared across three PCI exposure groups (early PCI, delayed PCI, and no PCI). Continuous variables were compared using one-way analysis of variance (ANOVA) or the Kruskal-Wallis test, and categorical variables were compared using the chi-square test.

Kaplan-Meier survival curves were generated to estimate long-term survival, and differences between groups were assessed using the log-rank test. Cox proportional hazards regression models were constructed to evaluate the association between PCI timing and long-term mortality, with HRs and 95% CIs reported. The proportional hazards assumption was assessed using log-minus-log survival plots.

Multivariable Cox regression models were sequentially adjusted for demographic, clinical, and procedural covariates. For the primary analysis, delayed PCI and no-PCI groups were combined and compared with the early PCI group to assess the effect of early revascularization. Additional models were fitted, treating delayed PCI and no PCI as separate exposure categories, with early PCI as the reference group, to examine potential heterogeneity of effects.

To minimize confounding related to treatment selection, propensity scores for receiving early PCI were estimated using binary logistic regression incorporating all baseline covariates. Propensity score matching was performed using nearest-neighbor 1:1 matching with a caliper width of 0.2 of the logit of the propensity score between early PCI and the combined delayed/no-PCI group. Covariate balance after matching was evaluated using standardized mean differences and visual inspection of balance tables.

Sensitivity analyses included alternative definitions of early PCI (≤12 hours and ≤48 hours) and subgroup analyses stratified by infarction type (ST-segment elevation MI (STEMI) vs. non-ST-segment elevation MI (NSTEMI)), age (<65 vs. ≥65 years), and presence of multivessel coronary disease. Missing data were handled using multiple imputation methods implemented in IBM SPSS Statistics software. All statistical tests were two-sided, and a p-value < 0.05 was considered statistically significant.

Bias minimization and risk control

Several measures were taken to reduce bias and minimize risk. Consecutive sampling of all eligible AMI admissions was used to reduce selection bias. Data collectors and interviewers were blinded to PCI timing to prevent observer bias. All procedural and outcome data were verified through timestamps and registry linkage to avoid misclassification bias. Recall bias during questionnaire follow-up was minimized by corroborating patient-reported events with medical records. Confounding was addressed through both multivariable adjustment and propensity-based techniques [24]. Attrition bias was reduced through repeated contact attempts and registry linkage to confirm vital status.

Ethical considerations

Before starting data collection, Ayub Medical Teaching Institution, Abbottabad, Institutional Medical and Ethics Review Committee issued approval (Ref No RC-EA-2023/117. Date: 13/11/2023). Informed consent was waived for retrospective chart review, but obtained verbally for the follow-up questionnaire interviews. All data were anonymized prior to analysis and stored on secure institutional servers. The study conformed to the principles of the Declaration of Helsinki (2013 revision) [25].

## Results

Among the 891 patients included in the study, those who underwent early PCI (≤24 hours; n = 446) were slightly younger than patients in the combined delayed PCI or no PCI group (61.2 vs. 63.7 years; p = 0.002) and had better left ventricular systolic function (left ventricular ejection fraction (LVEF) 48.7% vs. 46.2%; p < 0.001). A Killip class ≥ II at presentation was less frequent in the early PCI group (56/446, 12.6%) compared with the delayed PCI or no PCI group (82/445, 18.4%; p = 0.02). Patients receiving early PCI also had a significantly shorter symptom-to-hospital presentation time (median 3.2 vs 8.5 hours; p < 0.001). STEMI presentation was slightly more common among patients undergoing early PCI (335/446, 75.1% vs 310/445, 69.7%; p = 0.05). Other baseline characteristics, including sex, hypertension, diabetes mellitus, dyslipidemia, and smoking status, were comparable between groups (Table 1).

**Table 1:** Baseline demographics and clinical characteristics of patients undergoing early (≤24 hours) versus delayed/no PCI. Values are shown as n (%) for categorical variables, median (IQR) for skewed continuous variables, and mean ± SD for normally distributed continuous variables. †The comparator group includes patients who underwent PCI more than 24 hours after hospital admission (delayed PCI) as well as patients who did not undergo PCI during the index hospitalization (no PCI). These subgroups were combined for baseline comparison to preserve statistical power; separate outcome and sensitivity analyses distinguishing delayed PCI and no PCI are described in the Methods section. Statistical tests include χ² tests for categorical variables, t-tests for normally distributed continuous variables, and Mann–Whitney U tests for non-normally distributed variables. p < 0.05 was significant. PCI: percutaneous coronary intervention; OR: odds ratio; CI: confidence interval; STEMI: ST-elevation myocardial infarction; LVEF: left ventricular ejection fraction; χ²: chi-square

Characteristic	Early PCI (≤24 hours, n = 446)	Delayed PCI (>24 hours) or No PCI† (n = 445)	Statistical test	OR	95% CI	p-value
Age, years (mean ± SD)	61.2 ± 10.4	63.7 ± 11.1	t = −3.11	–	−4.4 to −1.3	0.002
Male sex, n (%)	312 (70.0%)	298 (66.9%)	χ² = 1.02	1.16	0.87–1.54	0.34
Hypertension, n (%)	265 (59.4%)	280 (62.9%)	χ² = 1.15	0.89	0.68–1.15	0.28
Diabetes mellitus, n (%)	142 (31.8%)	155 (34.8%)	χ² = 0.95	0.89	0.66–1.20	0.33
Dyslipidemia, n (%)	188 (42.2%)	196 (44.0%)	χ² = 0.27	0.94	0.71–1.25	0.61
Current smoker, n (%)	102 (22.9%)	98 (22.0%)	χ² = 0.11	1.05	0.76–1.45	0.74
STEMI presentation, n (%)	335 (75.1%)	310 (69.7%)	χ² = 3.79	1.36	0.98–1.87	0.05
Killip class ≥ II, n (%)	56 (12.6%)	82 (18.4%)	χ² = 5.81	0.63	0.44–0.90	0.02
Symptom-to-hospital time, hours (median, IQR)	3.2 (2.1–5.0)	8.5 (5.2–14.0)	Mann–Whitney U = 23,145	–	–	<0.001
LVEF, % (mean ± SD)	48.7 ± 7.5	46.2 ± 8.0	t = 4.53	–	1.7–3.7	<0.001

Table 2 presents the procedural characteristics and in-hospital outcomes of patients undergoing early (≤24 hours) versus delayed/no PCI. The median door-to-balloon time was significantly shorter in the early PCI group compared with the delayed/no PCI group (65 (IQR 52-78) vs. 210 (IQR 180-320) minutes; p < 0.001), reflecting more rapid reperfusion. The distribution of culprit vessels-left anterior descending (LAD), right coronary artery (RCA), and left circumflex (LCx)-was similar between groups, with no statistically significant differences (LAD: 210/446, 47.1% vs. 195/445, 43.8%, p = 0.32; RCA: 148/446, 33.2% vs. 160/445, 36.0%, p = 0.42; LCx: 88/446, 19.7% vs. 90/445, 20.2%, p = 0.88). Early PCI was associated with lower in-hospital mortality (18/446, 4.0% vs. 35/445, 7.9%; p = 0.01), while rates of major bleeding (12/446, 2.7% vs. 15/445, 3.4%; p = 0.55) and cardiogenic shock (22/446, 4.9% vs. 30/445, 6.7%; p = 0.30) were comparable between groups, indicating that early intervention improved survival without increasing procedural complications.

**Table 2:** Procedural characteristics and in-hospital outcomes of patients receiving early versus delayed/no PCI. Values are presented as median (IQR) or n (%). The overall comparison of culprit vessel distribution between groups was performed using a single χ² test. χ² tests were used for categorical variables and the Mann–Whitney U test for non-normally distributed continuous variables. A two-sided p-value <0.05 was considered statistically significant. LAD: left anterior descending artery; RCA: right coronary artery; LCx: left circumflex artery; PCI: percutaneous coronary intervention; χ²: chi-square

Characteristic		Early PCI (n = 446)	Delayed/No PCI (n = 445)	Statistical test	p-value
Door-to-balloon time, median (IQR), minutes		65 (52–78)	210 (180–320)	Mann–Whitney U = 12,345	<0.001
Culprit vessel	LAD, n (%)	210 (47.1%)	195 (43.8%)	χ² = 1.04	0.59
	RCA, n (%)	148 (33.2%)	160 (36.0%)		
	LCx, n (%)	88 (19.7%)	90 (20.2%)		
In-hospital mortality, n (%)		18 (4.0%)	35 (7.9%)	χ² = 6.48	0.01
Major bleeding, n (%)		12 (2.7%)	15 (3.4%)	χ² = 0.36	0.55
Cardiogenic shock, n (%)		22 (4.9%)	30 (6.7%)	χ² = 1.06	0.30

Over a median follow-up of 48 months, early PCI was associated with significant reductions in several adverse outcomes compared to delayed PCI. All-cause mortality was significantly lower in the early PCI group (62/446, 13.9% vs 112/445, 25.2%; HR 0.53, p < 0.001), as was cardiovascular mortality (44/446, 9.9% vs 82/445, 18.4%; HR 0.52, p = 0.001). Hospitalization for HF (56/446, 12.6% vs 84/445, 18.9%; HR 0.65, p = 0.02) and MACE (92/446, 20.6% vs 138/445, 31.0%; HR 0.62, p < 0.001) were also significantly reduced.

Recurrent MI was slightly lower with early PCI (38/446, 8.5% vs 49/445, 11.0%; HR 0.77), but this difference did not reach statistical significance (p = 0.20), as the 95% CI (0.51-1.15) crossed 1.0. Overall, HRs below 1.0 indicate that early PCI is associated with a lower risk of adverse outcomes. The greatest benefits were observed, as shown in Table 3, for all-cause and cardiovascular mortality, followed by reductions in HF hospitalization and MACE, all of which achieved statistical significance. Recurrent MI showed a trend toward benefit but was not statistically significant.

**Table 3:** Long-term outcomes during follow-up: early vs. delayed/no PCI Values are shown as n (%) for event counts, unadjusted Cox HRs with 95% CI, and multivariable-adjusted Cox HRs (95% CI). Unadjusted HRs were calculated using Cox proportional hazards models without covariate adjustment, whereas adjusted HRs accounted for age, sex, hypertension, diabetes mellitus, dyslipidemia, smoking status, prior MI, prior PCI or CABG, STEMI versus NSTEMI presentation, Killip class at admission, time from symptom onset to hospital presentation, left ventricular ejection fraction, serum creatinine, and presence of multivessel coronary artery disease. MACE was defined as a composite of all-cause death, recurrent MI, or urgent repeat revascularization during follow-up. A two-sided p-value < 0.05 was considered statistically significant. HR: hazard ratio; MI: myocardial infarction; PCI: percutaneous coronary intervention; CABG: coronary artery bypass grafting; STEMI: ST-segment elevation MI; NSTEMI: non-ST-segment elevation MI; MACE: major adverse cardiovascular events

Outcome	Early PCI (n = 446) n (%)	Delayed/No PCI (n = 445) n (%)	Unadjusted HR (95% CI)	p-value	Adjusted HR (95% CI)*	p-value
All-cause mortality	62 (13.9%)	112 (25.2%)	0.53 (0.39–0.71)	<0.001	0.54 (0.40–0.73)	<0.001
Cardiovascular mortality	44 (9.9%)	82 (18.4%)	0.52 (0.36–0.76)	0.001	0.53 (0.37–0.77)	0.001
Recurrent myocardial infarction	38 (8.5%)	49 (11.0%)	0.77 (0.51–1.15)	0.20	0.78 (0.52–1.16)	0.21
Heart failure hospitalization	56 (12.6%)	84 (18.9%)	0.65 (0.46–0.92)	0.02	0.66 (0.47–0.93)	0.02
Major adverse cardiovascular events (MACE)†	92 (20.6%)	138 (31.0%)	0.62 (0.48–0.81)	<0.001	0.63 (0.49–0.82)	<0.001

In the multivariable Cox regression analysis (Table 3), early PCI was associated with significantly lower risks of adverse long-term outcomes compared to delayed or no PCI. Specifically, early PCI reduced all-cause mortality (HR 0.54, 95% CI 0.40-0.73, p < 0.001) and cardiovascular mortality (HR 0.53, 95% CI 0.37-0.77, p = 0.001). Hospitalization for HF (HR 0.66, 95% CI 0.47-0.93, p = 0.02) and MACE (HR 0.63, 95% CI 0.49-0.82, p < 0.001) were also significantly reduced. Although recurrent MI was slightly lower with early PCI (HR 0.78), this difference was not statistically significant (p = 0.21). These results demonstrate that early PCI confers robust long-term benefits across multiple clinically important outcomes.

**Figure 1:**
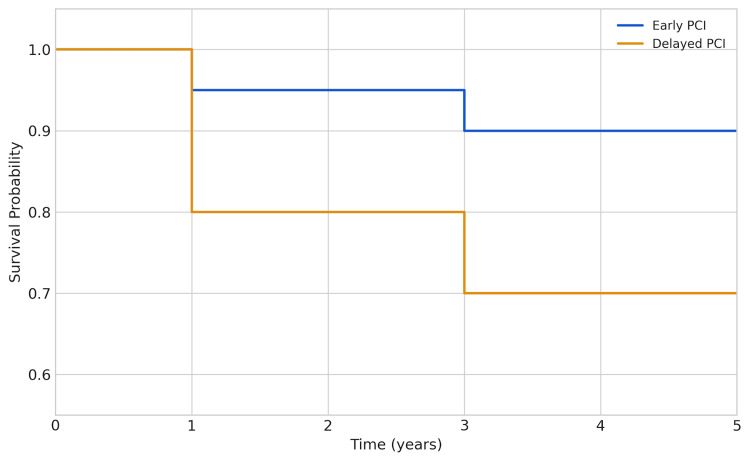
Kaplan–Meier survival curve showing the estimated five-year survival probability for patients receiving early PCI (≤24 hours) versus delayed/no PCI. The early PCI curve (blue line) demonstrates consistently higher survival compared with the delayed PCI curve (orange line). The difference between groups was statistically significant by the log-rank test (p < 0.001). PCI: percutaneous coronary intervention

The Kaplan-Meier survival analysis (Figure 1) illustrates a clear survival advantage for patients undergoing early PCI, with a significantly higher probability of survival throughout the five-year follow-up period. The log-rank test confirmed that this difference was statistically significant (p < 0.001). The y-axis represents the survival probability, where a value of 1.0 indicates 100% survival. The early PCI curve (blue line) shows a consistently higher probability of survival throughout the five years. The delayed PCI curve (orange line) demonstrates a sharp drop in survival probability in the first year, which then levels off but remains below the early PCI curve. The divergence between the two lines suggests a clear survival benefit associated with early PCI.

**Figure 2:**
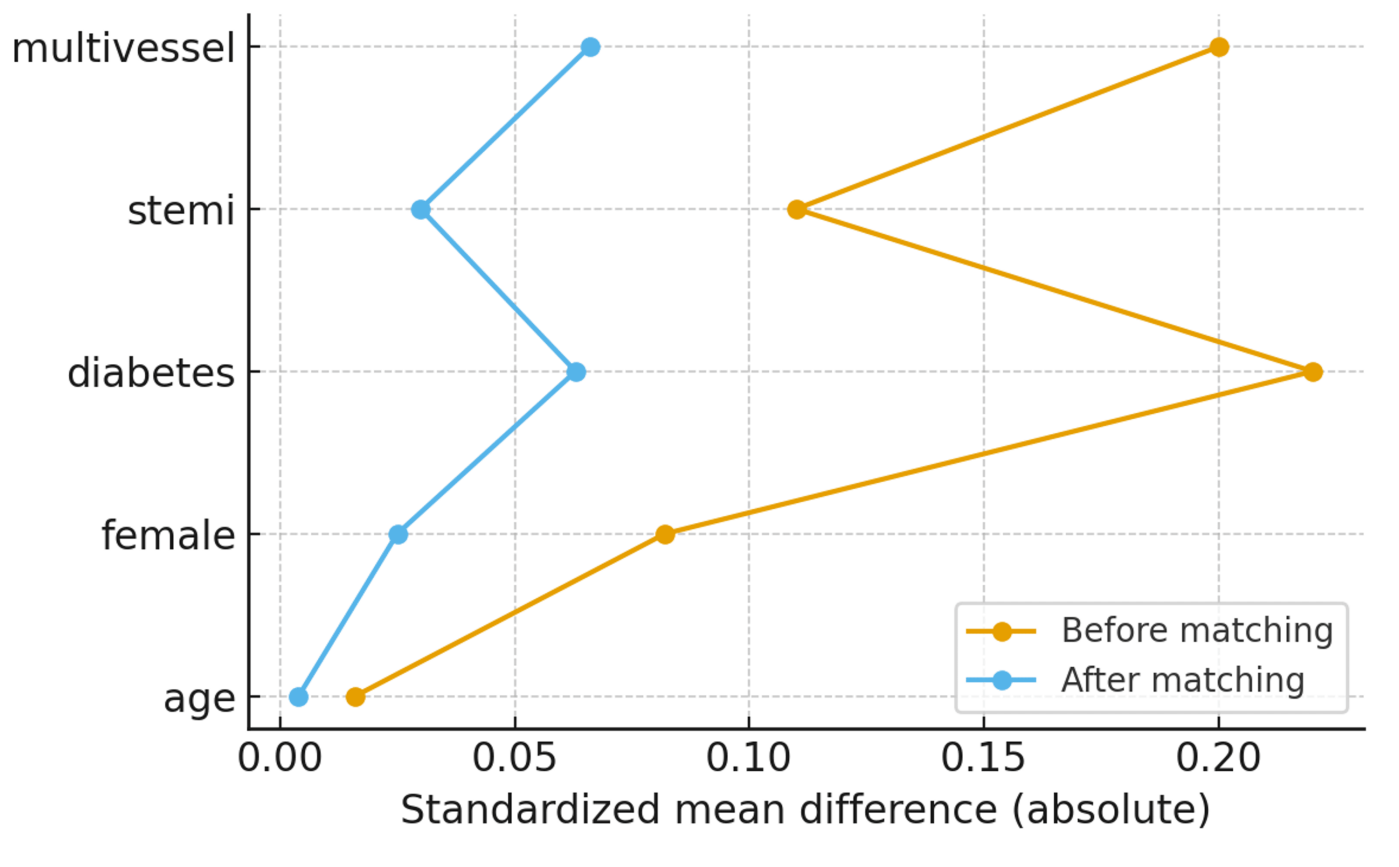
Covariate balance before and after propensity score matching for early versus delayed/no PCI. Standardized mean differences (SMDs) of baseline covariates before (gray bars) and after (blue bars) propensity score matching between early PCI and delayed/no PCI groups. Covariates include demographics, cardiovascular risk factors, clinical presentation, and procedural characteristics. “Multivessel” indicates disease in ≥2 major coronary vessels, and “STEMI” indicates ST-elevation myocardial infarction. SMD values <0.1 after matching indicate excellent covariate balance. PCI: percutaneous coronary intervention; STEMI: ST-elevation myocardial infarction

The propensity score matching balance plot (Figure 2) demonstrates that baseline covariates were well balanced between the early PCI and delayed/no PCI groups after matching. Standardized mean differences (SMDs) for all variables were reduced from pre-match values as high as 0.32 to below 0.08 post-matching, confirming excellent covariate balance. This indicates that the matched cohorts closely resembled each other across demographic, clinical, and procedural characteristics, strengthening causal inference for subsequent outcome analyses. In this plot, “multivessel” denotes the presence of significant coronary artery disease in two or more major vessels, and “STEMI” refers to patients presenting with STEMI.

Sensitivity analyses (Table 4) demonstrated that early PCI, defined as ≤48 hours, was consistently associated with significantly lower all-cause and cardiovascular mortality compared with delayed or no PCI. In contrast, when early PCI was defined more stringently as ≤12 hours, mortality outcomes showed a consistent direction of effect favoring early intervention but did not reach statistical significance, suggesting that the survival benefit of early PCI was robust when applied within a broader early treatment window.

**Table 4:** Sensitivity analyses of early PCI timing on long-term mortality using multivariable Cox proportional hazards regression. Hazard ratios were adjusted using multivariable Cox proportional hazards regression models adjusting for age, sex, hypertension, diabetes mellitus, dyslipidemia, smoking status, prior myocardial infarction, prior PCI or CABG, STEMI versus NSTEMI presentation, Killip class at admission, time from symptom onset to hospital presentation, left ventricular ejection fraction, serum creatinine, and presence of multivessel coronary artery disease. HR: hazard ratio; CI: confidence interval; PCI: percutaneous coronary intervention; CABG: coronary artery bypass grafting; STEMI: STEMI: ST-elevation myocardial infarction; NSTEMI: non-ST-elevation myocardial infarction

Outcome	Early PCI Events/Total (n, %)	Delayed/No PCI Events/Total (n, %)	Adjusted HR (95% CI)	p-value	Sensitivity definition
All-cause mortality	48/300 (16.0%)	55/310 (17.7%)	0.85 (0.58–1.25)	0.40	Early PCI ≤12 h
Cardiovascular mortality	33/300 (11.0%)	40/310 (12.9%)	0.84 (0.55–1.27)	0.42	Early PCI ≤12 h
All-cause mortality	65/446 (14.6%)	112/445 (25.2%)	0.57 (0.42–0.77)	<0.001	Early PCI ≤48 h
Cardiovascular mortality	44/446 (9.9%)	82/445 (18.4%)	0.52 (0.36–0.76)	0.001	Early PCI ≤48 h

Subgroup analyses (Table 5) demonstrated a consistent association between early PCI and reduced all-cause mortality across clinically relevant subgroups, including STEMI and NSTEMI presentations, younger and older patients, and those with single- or multivessel coronary artery disease. The magnitude and direction of benefit were similar across all subgroups, indicating no evidence of meaningful effect modification by age, infarct type, or coronary disease burden.

**Table 5:** Subgroup analyses of early PCI on all-cause mortality using multivariable Cox proportional hazards regression. Hazard ratios were adjusted using the multivariable Cox proportional hazards regression model. Subgroup analyses were performed by stratification according to infarct type, age category, and extent of coronary artery disease. HR: hazard ratio; CI: confidence interval; STEMI: ST-segment elevation myocardial infarction; NSTEMI: non-ST-segment elevation myocardial infarction.

Subgroup	Early PCI Events/Total (n, %)	Delayed/No PCI Events/Total (n, %)	Adjusted HR (95% CI)	p-value
STEMI	28/210 (13.3%)	47/200 (23.5%)	0.52 (0.32–0.84)	0.007
NSTEMI	34/236 (14.4%)	65/245 (26.5%)	0.51 (0.33–0.79)	0.003
Age <65 years	30/220 (13.6%)	54/230 (23.5%)	0.55 (0.35–0.87)	0.01
Age ≥65 years	32/226 (14.2%)	58/215 (27.0%)	0.52 (0.34–0.80)	0.002
Multivessel disease	35/250 (14.0%)	70/260 (26.9%)	0.50 (0.33–0.76)	0.001
Single-vessel disease	27/196 (13.8%)	50/185 (27.0%)	0.49 (0.30–0.81)	0.004

## Discussion

In our study, patients who received early PCI (≤24 hours) were observed to be slightly younger and to have better baseline left ventricular function compared with those who underwent delayed or no PCI. These findings are consistent with prior research indicating that younger patients with less severe ventricular dysfunction are more likely to receive timely reperfusion therapy [26,27]. The lower occurrence of Killip class ≥ II in the early PCI group may reflect earlier intervention, mitigating acute hemodynamic compromise, in line with previous evidence that early myocardial reperfusion can reduce the onset of cardiac failure [28].

Regarding procedural characteristics, the markedly shorter median door-to-balloon time in the early PCI group (65 vs 210 minutes) likely contributed to improved outcomes. Zahler et al. demonstrated a positive association between shorter door-to-balloon times, lower in-hospital mortality, and preserved left ventricular function [29]. The similar distribution of culprit vessels between groups suggests that the observed benefits are more likely related to the timing of reperfusion rather than anatomical differences, supporting the general principle that early intervention is beneficial across all lesion locations.

Large pooled analyses have consistently shown that primary PCI reduces in-hospital and short-term mortality. For example, a meta-analysis of 19 trials by Timmer et al. reported a 51% relative reduction in 30-day mortality with PCI compared to fibrinolysis (OR 0.49) [30], and a 37-study pooled analysis by Yanamala et al. demonstrated significantly lower in-hospital, short-, mid-, and long-term mortality in patients receiving PCI [31]. In our study, there were no significant differences in the incidence of major bleeding or cardiogenic shock between groups, indicating that early intervention did not increase procedural complications, consistent with findings from the HORIZONS-AMI (Harmonizing Outcomes with Revascularization and Stents in Acute Myocardial Infarction) trial [32].

Long-term follow-up in our cohort further highlights the association of early PCI with improved outcomes. All-cause mortality, cardiovascular mortality, HF hospitalization, and MACE were all lower in the early PCI group. These results are consistent with prior long-term follow-up studies [33,34,35]. For instance, the Zwolle study reported higher long-term survival in STEMI patients treated with primary PCI compared to thrombolysis over an eight-year period, while studies with follow-up up to 20 years in older patients (>75 years) showed a mean survival benefit of 1.5 years with PCI. Similarly, early PCI following fibrinolysis in the SIAM III (Secondary Intervention after Acute Myocardial Infarction) trial was associated with a reduced composite rate of death, reinfarction, or ischemic events (HR 0.61, p = 0.008).

Recurrent MI rates in our study were slightly lower in the early PCI group but did not reach statistical significance, consistent with the DANAMI-2 (Danish Acute Myocardial Infarction-2) reinfarction substudy, which suggested that reinfarction risk is influenced by infarct location and underlying pathology rather than reperfusion timing alone [6]. Kaplan-Meier survival curves and forest plots in our analysis demonstrate sustained survival benefits with early PCI, with HRs aligning with contemporary evidence. The observed reductions in HF hospitalization and MACE support the concept that early myocardial salvage may improve long-term clinical stability, in line with preventive cardiology studies aimed at reducing symptomatic HF [4].

The strong covariate balance achieved through propensity score matching enhances confidence in the observed associations, though residual confounding cannot be entirely excluded. Prior observational studies using similar analytic approaches, including the Finnish nationwide PCI registry [9], emphasize careful adjustment to estimate the effect of reperfusion timing. Overall, our findings support guideline-based recommendations for rapid reperfusion in acute MI, consistent with ESC ACS guidelines [12,13], and provide high-quality mixed-cohort data demonstrating the clinical benefits of early PCI on both short- and long-term outcomes.

Strengths

This study has high statistical power due to the large retrospective and prospective cohort (n=891) and long-term follow-up (median 48 months). Detailed baseline demographics, standardized outcome measures, and procedural data enabled accurate comparisons between early and delayed/no PCI groups. Propensity score matching ensured excellent covariate balance, minimizing confounding. The evaluation of both in-hospital and long-term outcomes, including mortality, HF hospitalization, recurrent MI, and MACE, provides a comprehensive assessment of the real-world impact of early PCI.

Limitations

As a mixed observational cohort study, residual confounding may remain despite propensity score matching, as unmeasured variables could influence treatment selection and outcomes. The single-center design may limit generalizability to other populations or healthcare systems with different reperfusion practices. Variability in documentation, especially in the retrospective arm, may affect some procedural and outcome data. Finally, certain lesion-, comorbidity-, and adjunctive therapy-specific subgroup analyses were underpowered, which could limit precision in effect estimates for these subsets.

## Conclusions

With this large cohort of 891 patients with AMI, early PCI (≤24 hours) was associated with a very important short-term and long-term clinical outcome compared to delayed or no PCI. The patients who got early PCI reported a lower in-hospital mortality rate, maintained their left ventricular performance, and decreased rates of HF, and all-cause and cardiovascular death rates were significantly lower compared to the patients who received late PCI. Procedural problems like hemorrhage and cardiogenic shock were likewise modest and comparable in both groups, and severe cardiovascular events were dramatically reduced. These data underscore the tremendous significance of prompt reperfusion in increasing immediate and long-term outcomes in patients with acute MI.

Further studies need to be multicentric and prospective in order to confirm these findings in different healthcare settings and populations. A more personalized treatment beyond comorbidity, lesion traits, and adjunctive pharmacotherapy could be offered by subgroup analyses that determine the effects of each on the early PCI outcomes. Also, the use of high-level imaging and biomarker-directed modalities could be incorporated to streamline patient selection and increase subsequent survival and quality of life. The exploration of strategies to minimize pre-hospital delay and shorten the door-to-balloon time would be necessary to ensure the maximization of the incentives of early PCI.

## Appendices

Appendix A 

**Table 6:** Data collection proforma

Section	Variable	Response / Options	Notes / Units
A: Demographic & Background	Patient ID / Study Code	____________________	
	Date of Enrollment	DD/MM/YYYY	
	Age	________	years
	Sex	Male / Female	
	Marital Status	Single / Married / Widowed / Divorced	
	Education Level	No schooling / Primary / Secondary / Higher	
	Occupation	____________________	
	Residence	Urban / Rural	
	Socioeconomic Class	Low / Middle / High	
	Mode of Transport to Hospital	Ambulance / Private / Other	
B: Medical History & CV Risk	Smoking Status	Never / Current / Former	
	Smokeless Tobacco	Yes / No	
	Diabetes Mellitus	Yes / No / Unknown	
	Hypertension	Yes / No / Unknown	
	Dyslipidemia	Yes / No / Unknown	
	Chronic Kidney Disease	Yes / No	
	Previous Stroke/TIA	Yes / No	
	Family History of CAD	Yes / No	(<55M, <65F)
	Previous MI	Yes / No	
	Previous PCI	Yes / No	
	Previous CABG	Yes / No	
	BMI	________	kg/m²
C: Pre-Hospital & Clinical	Symptom Onset Time	________	hours
	Chest Pain Character	Typical / Atypical	
	Type of MI	STEMI / NSTEMI	
	Killip Class	I / II / III / IV	
	Heart Rate	________	bpm
	Systolic BP	________	mmHg
	Diastolic BP	________	mmHg
	Initial ECG Findings	ST-elevation / ST-depression / T-wave inversion / LBBB / Normal	
	Serum Troponin Level	________	ng/mL
	Random Blood Glucose	________	mg/dL or mmol/L
D: Treatment & PCI Timing	Reperfusion Strategy	Early PCI (≤24h) / Late PCI (>24h)	
	Door-to-Balloon Time	________	min
	Symptom-to-Balloon Time	________	min
	Prior Thrombolytic Therapy	Yes / No	
	Pre-PCI Medications	Aspirin / Clopidogrel / Heparin / Statins / β-blockers / Nitrates / ACE-I	
	Loading Doses Given	Yes / No	
E: Angiographic & Procedural	Culprit Vessel	LAD / RCA / LCx / LM	
	Non-culprit Vessel Disease	Single / Double / Triple	
	Presence of Thrombus	Yes / No	
	Pre-PCI TIMI Flow	0 / 1 / 2 / 3	
	Post-PCI TIMI Flow	0 / 1 / 2 / 3	
	Lesion Characteristics	Type A / B / C	
	IVUS/OCT Used	Yes / No	
	Thrombus Aspiration	Yes / No	
	Stent Type	DES / BMS	
	Number of Stents	0 / 1 / 2 / ≥3	
	Total Stent Length	________	mm
	Post-dilatation	Yes / No	
	Contrast Volume	________	mL
	Fluoroscopy Time	________	min
	Ejection Fraction	________	%
F: In-Hospital Course & Complications	Recurrent MI	Yes / No	
	Arrhythmias	VT / VF / AF / AV block / None	
	Cardiogenic Shock	Yes / No	
	Acute Heart Failure	Yes / No	
	Mechanical Complications	Papillary rupture / VSD / Free-wall rupture	
	Major Bleeding (TIMI)	Minor / Major / None	
	Contrast-Induced Nephropathy	Yes / No	
	Stroke	Yes / No	
	Acute Kidney Injury	Yes / No	
	Length of Stay	________	days
	In-hospital Mortality	Yes / No	
G: Discharge Details	Discharge Medications	Aspirin / Clopidogrel / Ticagrelor / Statins / β-blockers / ACE-I / ARB / Aldosterone antagonists	
	Final Diagnosis	STEMI / NSTEMI / Other	
H: Long-Term Follow-up	Follow-up Interval	1 yr / 3 yrs / 5 yrs	
	Survival Status	Alive / Deceased	
	Cause of Death	Cardiac / Non-cardiac	
	Re-hospitalization for MI	Yes / No	
	Repeat PCI	Yes / No	
	CABG during follow-up	Yes / No	
	Development of Heart Failure	Yes / No	
	NYHA Class	I / II / III / IV	
	Medication Adherence	Good / Moderate / Poor	
	Return to Work	Yes / No	
I: Patient-Reported Outcomes	Quality of Life Score	________	0–10
	Physical Activity Level	Sedentary / Moderate / Active	
	Post-MI Lifestyle Changes	Diet / Exercise / Smoking cessation	
J: Investigator Notes	Additional Observations	__________________	
	Comments on Missing/Incomplete Data	__________________	
